# Validation of Lyophilized Human Fecal Microbiota for the Treatment of *Clostridioides difficile* Infection: A Pilot Study with Pharmacoeconomic Analysis of a Middle-Income Country—Promicrobioma Project

**DOI:** 10.3390/microorganisms12081741

**Published:** 2024-08-22

**Authors:** Carolina Hikari Yamada, Gabriel Burato Ortis, Gustavo Martini Buso, Thalissa Colodiano Martins, Tiago Zequinao, Joao Paulo Telles, Luciana Cristina Wollmann, Carolina de Oliveira Montenegro, Leticia Ramos Dantas, June Westarb Cruz, Felipe Francisco Tuon

**Affiliations:** 1 Laboratory of Emerging Infectious Diseases, School of Medicine, Pontifícia Universidade Católica do Paraná, Curitiba 80215-901, PR, Brazil; carolina.yamada@huemackenzie.org.br (C.H.Y.); gabriel.burato@pucpr.edu.br (G.B.O.); thalissa.cmartins@ter.grupomarista.org.br (T.C.M.); zequinao.tiago@hospitalcajuru.com.br (T.Z.); leticia.dantas@pucpr.edu.br (L.R.D.); 2 School of Business, Pontifical Catholic University of Paraná, Rua Imaculada Conceição 1155, Curitiba 80215-901, PR, Brazil; gustavo.buso@pucpr.br (G.M.B.); june.cruz@pucpr.br (J.W.C.); 3 Hospital Universitário Evangélico Mackenzie, Curitiba 80730-150, PR, Brazil; jpmarochi@hotmail.com (J.P.T.); cristina.wollmann@huemackenzie.org.br (L.C.W.)

**Keywords:** *Clostridium difficile*, fecal microbiota transplantation, cost-effective, antibiotics, public health

## Abstract

Background: *Clostridioides difficile* infection (CDI) represents a prevalent and potentially severe health concern linked to the usage of broad-spectrum antibiotics. The aim of this study was to evaluate a new lyophilized product based on human fecal microbiota for transplant, including cost–benefit analysis in the treatment of recurrent or refractory CDI. Methods: The product for fecal microbiota transplant was obtained from two donors. Microbiological, viability, and genomic analysis were evaluated. After validation, a clinical pilot study including recurrent or refractory CDI with 24 patients was performed. Clinical response and 4-week recurrence were the outcome. Cost–benefit analysis compared the fecal microbiota transplant with conventional retreatment with vancomycin or metronidazole. Results: The microbiota for transplant presented significant bacterial viability, with and adequate balance of Firmicutes and Bacteroidetes. The clinical response with the microbiota transplant was 92%. In financial terms, estimated expenditure for CDI solely related to recurrence, based on stochastic modeling, totals USD 222.8 million per year in Brazil. Conclusions: The lyophilized human fecal microbiota for transplant is safe and can be an important step for a new product with low cost, even with genomic sequencing. Fecal microbiota transplantation emerges as a more cost-effective alternative compared to antimicrobials in the retreatment of CDI.

## 1. Introduction

Infections caused by *Clostridioides difficile* (CDI) represent the predominant etiology of infectious diarrhea among hospitalized patients [1]. Such occurrences are notably heightened in individuals who have used antimicrobial therapy, particularly those medications with impact on the gut microbiota, thereby fostering a dysbiosis pathway ending in proliferation of *C. difficile* [2]. Consequently, its incidence demonstrates a direct correlation with the indiscriminate utilization of antimicrobials [2]. Recognized as a nosocomial infection, CDI increases morbimortality rates within patient cohorts [1]. For instance, at the end of 2017 in the United States, there were 223,900 CDI cases and 12,800 fatalities among hospitalized patients [3].

In the context of developing nations, there is a dearth of data regarding (i) the real prevalence of CDI, (ii) the predominant circulating strains, and (iii) the economic cost associated with the lack of disease control [4]. Moreover, diagnostic challenges abound, not only due to the lack of lab resources but also the economic burden imposed by the ideal diagnosis pathway (e.g., multistep algorithm). Molecular tests are infrequently conducted due to the limited number of laboratories equipped to perform such specialized diagnostics. Consequently, the prevalence of CDI remains underreported within national databases. Despite previous data regarding the costs of CDI, mainly to health public systems in high-income regions [5,6], there is still a need got data from health economics and outcomes research (HEOR) to better elucidate the cost-effectiveness of the varied strategies for CDI diagnosis, treatment, and prevention.

The major therapeutic modalities for CDI are metronidazole and/or vancomycin over a period of 10 to 14 days. Nevertheless, primary treatment failure rates can reach 40%, which may prompt consideration of a fecal microbiota transplantation (FMT) [7]. The recurrence or secondary failure after a second course of antibiotics remains even higher, with rates reaching 60% [8]. Considering that FMT has exhibited notable efficacy in CDI treatment, there is a heightened interest in exploring its therapeutic potential and impact on clinical outcomes [8]. Fecal microbiota transplantation aims to address dysbiosis by modifying the recipient’s microbiome through introducing a healthy donor’s microbiota [9]. This procedure, particularly effective in cases of recurrent CDI (rCDI), disrupts the environment that *C. difficile* thrives in. Studies have shown that fecal microbiota transplantation leads to a notable reduction in dysbiosis and an enhancement in gut microbial diversity among individuals with rCDI [10]. However, time and financial constraints associated with fecal microbiota preparation (e.g., donor screening and selection, stool preparation, freezing or lyophilization methods) and the logistical challenges in accessing this therapy (e.g., frozen transportation) highlight the call to gauge the cost-effectiveness within the health-care systems of developing countries. Fecal microbiota transplantation offers advantages over antibiotic therapy, including earlier cessation of diarrhea, potential for shorter hospital stays, reduced antimicrobial usage, early improvement of dysbiosis, and decreased duration of contact isolation. One of the challenges with FMT in most countries, including developing nations, is the interpretation of FMT as either a product or a service for processing human samples. There is no specific legislation for either case, and regulatory agencies, such as ANVISA (the National Health Surveillance Agency) in Brazil, do not have a position on these proposals either and are unwilling to take a stance. Additionally, there is a need for specialized teams to ensure that all safety measures are adequately implemented. These factors collectively suggest that FMT may confer greater benefits than retreatment with antibiotics; however, a cost–benefit analysis is warranted. The objective of this study is to validate a product based on human fecal microbiota for transplantation in patients with CDI, and to present a cost–benefit analysis to define its viability and value in developing countries.

## 2. Methods

This research was developed in three steps: (1) obtaining and validating human fecal microbiota for transplant; (2) pilot clinical study; (3) cost–benefit evaluation. The Promicrobioma Project was initiated in 2020 to develop a human fecal microbiota bank for transplantation in patients with CDI. Over the course of two years, the research focused on validating techniques, establishing physical infrastructure, and training staff to ensure the development of a service for processing and creating a product according to best practices.

### 2.1. Human Fecal Microbiota for Transplant

#### 2.1.1. Donor Selection and Testing

The product used for FMT comes from 2 fixed donors. The selection process involved recruitment and pre-evaluation stages, followed by clinical and laboratory tests, including microbiota analysis through next-generation sequencing. For donor selection, we followed previous protocols [9,11]. The questionnaire and tests conducted were in accordance with the international consensus conference on stool banking for fecal microbiota transplantation in clinical practice [12].

#### 2.1.2. Feces Processing

**Figure 1:**
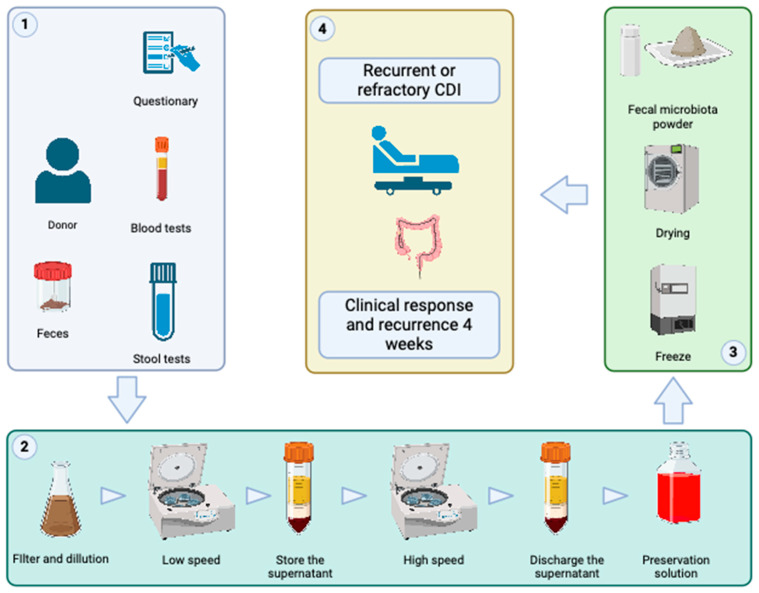
Steps of fecal microbiota preparation for transplant. ① Donor screening; ② microbiota processing; ③ Lyophilization and Storage; ④ Indication of microbiota transplant and follow-up.

Initially, the processing of microbiota begins with weighing the fecal material received from the donor. The feces are then transferred to a container with a ratio of 250 mL of saline solution for every 50 g of feces. This mixture is homogenized for 2 to 5 min. The suspension is filtered twice through a filter made of 5 layers of gauze. After filtration, the content is centrifuged at 200× *g* for 10 min. The supernatant after centrifugation is transferred through filtration with 5 layers of gauze into a sterile 50 mL conical tube. The remaining solid residue is discarded. The solution is then centrifuged again at 4200× *g* for 15 min. The supernatant is discarded, and a cell preservation solution (in the patenting process) is added. The samples undergo freeze-drying for lyophilization. In powdered form, the microbiota is then stored until it reaches 320 g, at which point it is divided into 32 g fractions, which are the equivalent used for each transplant. The stored samples always originate from the same donor (Figure 1).

#### 2.1.3. Non-Bacterial Fecal Residue Analysis

Qualitative scanning electron microscopy (SEM) sample preparation followed a previously described protocol [13,14]. One gram of the final product was spread in a sterile glass slide and filled with primary fixative agent (0.68 g^−1^ sucrose, 0.42 g^−1^ sodium cacodylate, 0.6 mL^−1^ 30% glutaraldehyde) (Merck, Darmstadt, Germany) and 19.4 mL^−1^ of deionized water, fully covering specimens for 45 min. The slide was transferred to a buffer (containing sucrose and sodium cacodylate in the same concentrations as the fixative agent) for 10 min. Afterwards, specimens were dehydrated in an increasing ethanol series (35%, 50%, 70%, and 100%), followed by 100% hexamethyldisilane (HMDS) (Merck, Darmstadt, Germany) for fixation. Each step lasted 10 min. The slide was then coated with gold particles in a metalizing instrument with a Q150R ES rotary pump (Quorum Technologies, Lewes, UK), and fixed on a metal base for SEM observation under a PentaFET Precision (Oxford Instruments, Abingdon, UK) at 5.0 kV. Observations were made at magnifications between 2000× and 6000×, looking for materials other than bacteria. The tests were performed in quintuplicate and from samples at 3 different times. A control group was included without the processing.

#### 2.1.4. Bacterial Viability and Shelf-Life

For the viability test, 1 g of the processed material was diluted in 1 mL of ultrapure water, and from this solution, progressive dilutions were made for quantitative analysis of colony-forming units (CFUs) per gram of processed fecal microbiota. Cultures were performed on horse blood agar, chocolate agar, and MacConkey agar under aerobic and anaerobic conditions (triplicate). The counting was done manually, and we considered the agar plate with larger CFU/g for counting. The test was conducted on day 1 post-lyophilization and then at 1, 6, 12, and 24 months. The processed and lyophilized material was stored in a refrigerator at 5–8 °C for constant temperature control purposes.

Five samples from the processed fecal microbiota were allocated into sterile 24-well plates with a previously used 13 mm rounded glass coverslip (Sarstedt, Newton, NC, USA) on the bottom of each well and stained with a FilmTracer™ LIVE/DEAD^®^ Biofilm Viability kit (Molecular Probes, Life Technologies Ltd., Paisley, UK), aiming to determine the viability of bacteria. The coverslips were immediately subjected to fluorescence microscopy (Zeiss Scope. A1; Carl Zeiss, Oberkochen, Germany). All the specimen samples were stained and treated following the kit protocol, using 480/500 nm excitation/emission for SYTO^®^ 9 and 490/635 nm excitation/emission for propidium iodide [15]. A group of samples frozen at −20 °C for 24 h followed by defrosting was used to compare with the lyophilization method.

##### Next-Generation Sequencing

DNA was extracted using DNeasy PowerSoil (Qiagen, Hilden, Germany) and quantified with NanoDrop (Thermo, Waltham, MA, USA). The V3V4 region of the 16S rDNA was amplified with primers 341F and 805R, both with Nextera barcodes according to the manufacturer’s instructions. The amplicons were quantified with a Quantus DNA kit (Promega, Madison, WI, USA) and sequenced with a P1-600 kit (Illumina, San Diego, CA, USA) on a NextSeq 1000 (Illumina) in paired-end 2 × 300 bp mode. Sequencing data were processed using QIIME2 (v.2022.2) [16]. Sequences were pre-processed, including removal of low-quality sequences, sequencing error correction, chimera removal, and identification of amplicon sequence variants (ASVs). For this, we used the DADA2 method with default parameters. Taxonomy was assigned to the ASVs using the naïve Bayes approach implemented via the scikit-learn Python library with default parameters and the GTDB (v. 207) database for bacteria.

16S_341F TCGTCGGCAGCGTCAGATGTGTATAAGAGACAGCCTACGGGNGGCWGCAG

16S_805R GTCTCGTGGGCTCGGAGATGTGTATAAGAGACAGGACTACHVGGGTATCTAATCC

Library preparation, sequence analysis, and bioinformatics were performed as previously described [17].

### 2.2. Pilot Clinical Study

#### 2.2.1. Study Design

This was a pilot study without a comparator group to evaluate the safety and clinical response of patients with CDI, including refractory or recurrent cases, using our product for FMT (Promicrobioma Project).

#### 2.2.2. Setting

The study was approved by the Human Research Ethics Committee of the Pontifical Catholic University of Paraná under protocol 51343521.3.0000.0020. The study was conducted at 2 Brazilian hospitals located in Curitiba.

#### 2.2.3. Participants

Consecutive patients (convenience sample) with either refractory CDI or recurrence were included in the study. All patients had a confirmed diagnosis of CDI by toxin A/B + glutamate dehydrogenase testing OR molecular testing + glutamate dehydrogenase. Patients were required to be symptomatic at the time of transplantation indication. Refractory patients were defined as those who had received two different antibiotic regimens consecutively, each for at least 10 days. The treatments considered for CDI included vancomycin or metronidazole, which are the only medications available in Brazil. Recurrence cases included patients who had a new CDI diagnosis within 28 days after completing antimicrobial treatment. Patients were not included if they were being treated for recurrence prevention, required antibiotics for other purposes, had active infections other than CDI, were pregnant, or were under 18 years of age.

#### 2.2.4. Intervention

The patients were informed about the risks and benefits of the procedure by their attending physician and signed a consent form. The lyophilized microbiota was provided in 2 vials, with the material to be diluted in 80 mL of saline solution (0.9% NaCl). The vial was shaken until a homogeneous solution was obtained, then the material was aspirated for infusion. Conventional bowel preparation was routinely performed before colonoscopy. The microbiota was administered into the right colon. Patients were instructed to try and retain the transplanted material for at least 30 to 40 min, but preferably for more than 4 h. For this, 1 to 2 tablets of loperamide were prescribed immediately after the transplant and again after 6 h.

#### 2.2.5. Variables

Epidemiological data such as age and sex, clinical comorbidities, prior antibiotic use, and previous treatments for CDI were evaluated. The use of immunosuppressants or organ transplantation was also assessed. The number of bowel movements and their duration before and after the transplant were evaluated. The primary outcome variable was clinical improvement, with the secondary outcome being recurrence within 4 weeks as previously described [18].

#### 2.2.6. Data Sources and Study Size

Clinical data were obtained directly from medical records and through structured interviews. Follow-up data were also collected via structured questionnaires and medical evaluations at 48 h post-procedure, 1 week after, and at 30 days. The study was designed as a pilot, and considering that the medical literature reports a clinical cure rate of >90% and a decrease in recurrence with FMT, we estimated that at least 20 patients would be sufficient for analyzing the safety of the lyophilized microbiota, as well as evaluating clinical cure and recurrence.

### 2.3. Cost-Effectiveness Evaluation

#### 2.3.1. Study Design

This research adopted a predictive, descriptive, and exploratory approach, utilizing primary data sources. Data such as health expenditure were obtained from two medium-sized hospitals in a developing country, specifically chosen as Brazil, while demographic and CDI incidence data serving as the model’s foundation were sourced from the Decennial Census and the Centers for Disease Control and Prevention of the United States. This approach enabled the construction of a comprehensive overview of CDI, facilitating the estimation of CDI incidence and associated treatment costs.

#### 2.3.2. Setting

The two selected hospitals are situated in Curitiba, southern Brazil. One of the hospitals serves as a trauma and emergency reference, encompassing orthopedics and neurosurgery, with a total of 207 beds, including 29 intensive care unit (ICU) beds. The other hospital specializes in clinical, oncological, and elective surgeries, boasting 183 beds, including 60 ICU beds.

#### 2.3.3. Data Source

The initial study phase involved obtaining and parameterizing demographic data for both the United States of America and Brazil. The total population in each region was documented, stratified by sex and age group, and sex ratio analysis conducted for both populations. In the subsequent phase, public data on CDI from the Emerging Infections Program (EIP) of the CDC covering the period from 2013 to 2021 were collected and structured. The same sex and age stratification used in the first phase was applied. Cost-related data were acquired from the electronic medical systems of the two hospitals included in the study, involving a detailed analysis of fixed and variable costs.

#### 2.3.4. Variables

The diagnosis of CDI requires meeting two conditions: (a) the existence, of symptoms compatible with the infection, ruling out any other potential cause, generally the presence of diarrhea (3 or more unformed stools in the last 24 h); and (b) the detection of toxins A and/or B or toxigenic C [19]. Based on the EIP CDC data, the analysis included the total number of CDI cases and incidence per 100,000 inhabitants, calculated using the formula ((number of cases/monitored population) × 100,000). This approach allowed for the assessment of CDI cases in both the community and health-care settings, along with the overall case count. CDI cases were classified as new or recurrent based on the CDC guidelines for CDI diagnosis. Variable and fixed costs associated with CDI hospitalization, antimicrobial treatment, and fecal microbiota transplantation were analyzed and expressed in US dollars. The cost of fecal microbiota transplantation was calculated based on the fecal microbiota bank at the Catholic University of Paraná, which conducts transplants for Brazil (USD 839.54). This value considered costs of colonoscopy and all consumables and technical workload from the fecal microbiota bank, in the same locale where the lyophilized microbiota was developed, as described before.

### 2.4. Statistical Analysis

The data from this study were non-comparative, and therefore no statistical treatment was applied for the pilot study. Quantitative data are described using medians and interquartile ranges and qualitative data with percentages. The validation phase data were either descriptive or quantitative without comparative analysis. For the cost-effectiveness study, a database analysis from the United States was used to assess incidence, as epidemiological data in Brazil were insufficient.

With structurally equivalent databases, the analysis proceeded to examine the proportions of cases in the United States and Brazil using the simple rule of three expressed by the formula:

$$\frac{x1}{x2}=\frac{y1}{y2}$$

To conduct incidence projections, the Monte Carlo simulation technique was adopted with 10,000 simulations. This approach involves generating scenarios from a specific probability distribution and using the resulting sample to approximate the function of interest. This allows for the simulation of a wide range of situations and possible outcomes, as expressed by the formula:

ds = µ s dt + σ s dz

Subsequently, the Monte Carlo simulation was again employed to create specific scenarios of incident cases in the community and health-care settings, confirming the earlier stochastic analysis, with a margin of error of 5% and repeated 1000 times.

After that, the data were combined: (i) the analyses of the quantity of incident cases of CDI in the community and health-care settings and (ii) the costs from the hospitals in Curitiba and the treatments covered by the Brazilian Health System (SUS). Therefore, it was possible to analyze the costs related to available treatments and determine the effectiveness and/or financial benefit of each treatment. Finally, using recurrence data provided by the CDC, a projection was made for the Brazilian scenario and multiplied by costs, considering recurrent cases in wards and ICUs, resulting in the estimated financial amount for spending in Brazil on recurrent CDI.

## 3. Results

### 3.1. Fecal Microbiota Validation for Transplant

**Figure 2:**
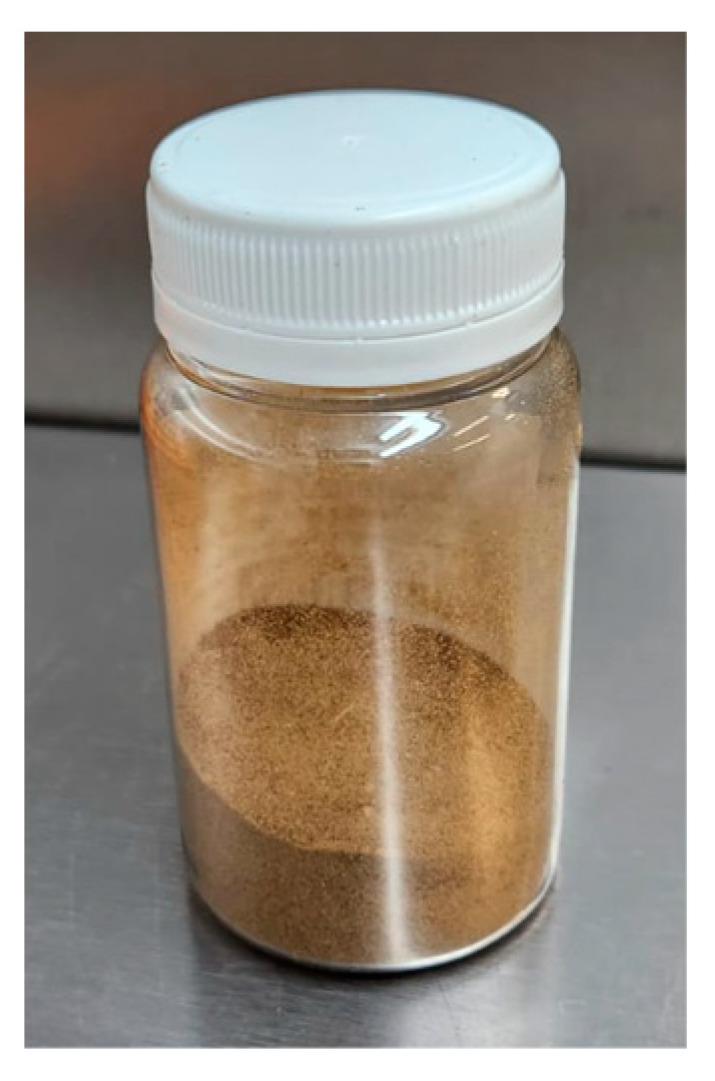
Lyophilized fecal microbiota preparation for transplant.

**Figure 3:**
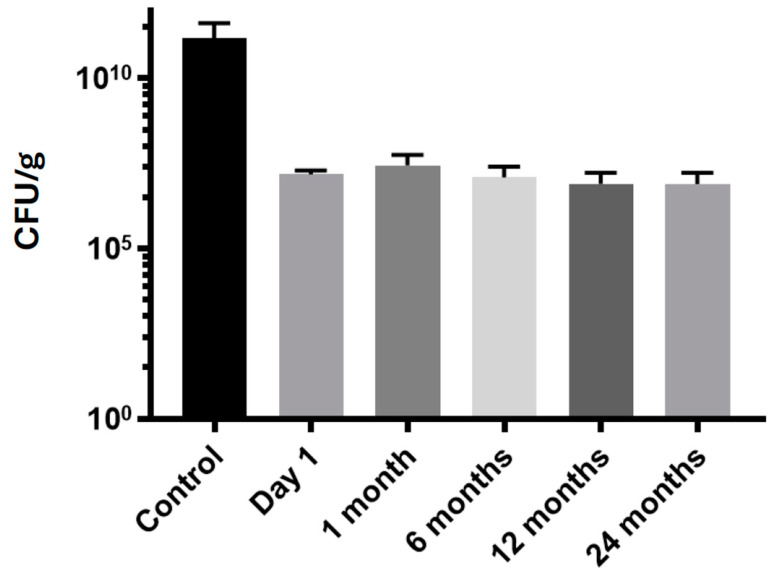
Quantification of fecal microbiota in agar plate culture in natura (control) and after lyophilization.

The final sample was a fine brownish powder, as shown in Figure 2. The cell viability test through culture demonstrated a reduction in the total quantity of bacteria in the range of two logarithms of 10, using the feces before processing as a control (Figure 3). The control value found was 1.8 × 10^9^ (IQR 7.5 × 10^7^–4.2 × 10^11^) CFU/g, and on day 1 of lyophilization, it was 1.9 × 10^7^ CFU/g (1.0 × 10^7^–1.9 × 10^7^). At the 2-year evaluation, the value was 6.6 × 10^6^ CFU/g (1.2 × 10^5^–1.6 × 10^7^).

**Figure 4:**
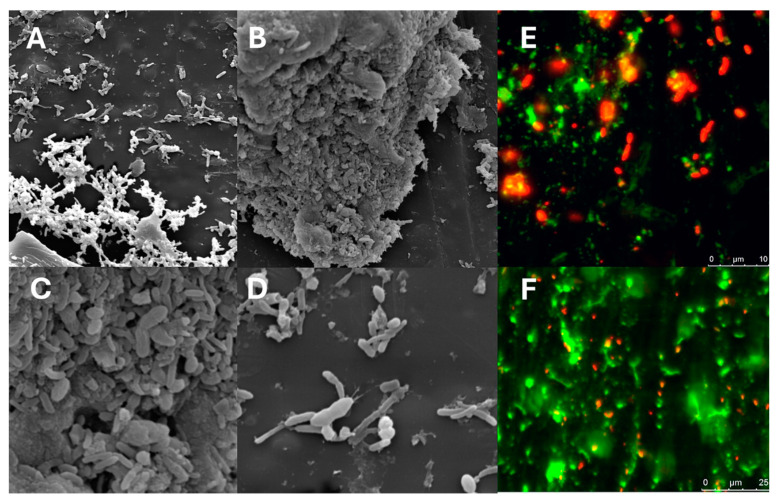
Evaluation of lyophilized human fecal microbiota using scanning electronic microscopy (SEM) for fecal residues. The scanning presented few residues ((**A**) 10,000×; (**B**) 10,000× showing a conglomerate of bacteria; (**C**) 50,000× in the conglomerate; and (**D**) 50,000× in a free area). The bacterial viability test using fluorescence showing significant viability after lyophilization (**F**), freezing at −20 °C, and defrosting (**E**).

With a Live/Dead^®^ viability kit, significant bacterial viability was demonstrated—>95% (Figure 4). Scanning electron microscopy (SEM) evaluation of residues demonstrated that the major component of the material was composed of bacteria, with a small number of aggregates corresponding to clusters of bacteria with components of the cell preservation solution. No other residues such as fibers, food remnants, or contaminants were found (Figure 4).

Next-generation sequencing demonstrated a good Firmicutes-to-Bacteroidetes ratio (approximately 95% in donor 1 and 92% in donor 2). No pathogenic bacteria were found using an identification limit of up to 0.01%. All microorganisms detected in both donors throughout the study are described in the Supplementary Materials.

### 3.2. Pilot Clinical Study

For the 24 patients included in the clinical study, the mean age was 65 ± 11 years (Table 1). Twenty patients had some comorbidity, including one with advanced HIV and two with renal transplants. Diagnosis was made using two tests (PCR + GDH/toxin) or just GDH + toxin. FMT was indicated in 11 cases of recurrence (46%) and 14 cases of antibiotic refractoriness (54%). The average number of bowel movements pre-FMT was 8 ± 4 episodes per day. Clinical response was achieved in 92% of the patients, with two failures. In one of the clinical failures, the patient received systemic antibiotics during the procedure (meropenem with linezolid). No patient reported any adverse events related to the transplant.

### 3.3. Cost–Benefit Evaluation

The initial analysis constructed was demographic, considering the total population of the United States and Brazil. For the U.S., a total of 331.45 million people were considered based on the 2022 census, with 49.08% male and 50.91% female. As for Brazil, the population according to the 2022 census was 203.06 million, with 48.89% male and 51.11% female. When stratifying populations by age group, the proportion of USA people ≥65 years is 1.63 times larger than the Brazilian proportion (USA: 16.83% vs. Brazil: 10.30%).

**Figure 5:**
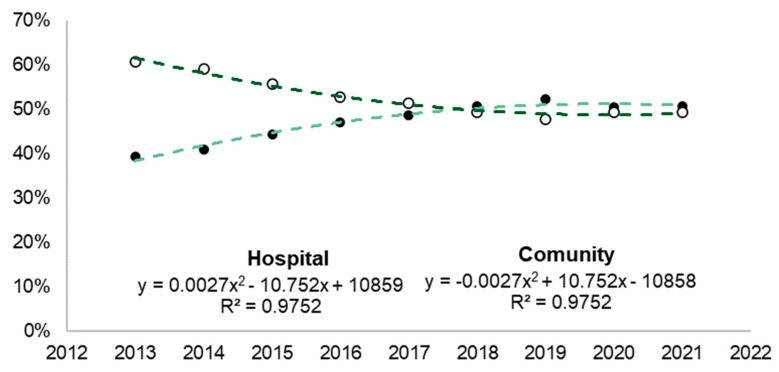
Case incidence rate in the population monitored by the CDC. White circles represent cases in health care, black circles represent cases in the community, and green lines represent polynomial trends.

Table 2 presents the total number of CDI cases per 100,000 inhabitants per year in the United States from 2013 to 2021. Considering the total period and age distribution, 21.42% were under 18 years old, 40.06% were between 18 and 44 years old, 24.01% were between 45 and 64 years old, and 14.51% were 65 years or older. Additionally, it is possible to analyze the incidence rates of CDI in the population using a polynomial trend line (Figure 5). Data demonstrated that there was a decreased incidence of health-acquired CDI (HA-CDI) (R = 0.9752) and an increase in community-acquired CDI (CA-CDI) (R = 0.9752).

After a comprehensive analysis of the data monitored by the CDC in various counties, we can establish an overview of the situation of CDI in Brazil (Table 3). Using the Monte Carlo simulation method, we identified that there are 273,957 Brazilian cases per year, with a variation of σ ± 7931, representing an incidence of approximately 0.1353%. From this analysis, about 40% (*n* = 111,043) occur in the community, while the remaining 60% (*n* = 162,914) are related to health-care settings. Considering gender segmentation, 59.76% (*n* = 163,723 cases (σ ± 4623)) occur among females, while 40.23% (*n* = 111,903 cases (σ ± 3961)) affect males.

When evaluated, incidence per 100,000 population CA-CDI was more common in people < 45 years (CA-CDI (*n* = 35,657; 26 cases/100,000 pop.) vs. HA-CDI (*n* = 20,556; 15 cases/100,000 pop.), while HA-CDI was more probable in people > 45 years (CA-CDI (*n* = 70,835; 105 cases/100,000 pop.) vs. HA-CDI (*n* = 146,909; 218 cases/100,000 pop.). Nevertheless, considering the total CDI cases, there were significant differences regarding the distribution by age group when comparing CA and HA-CDI, respectively: (i) aged 1–17 years 7.89% (*n* = 8411) vs. 1.9% (*n* = 3285) (*p* < 0.00001); (ii) aged 18–44 years 25.58% (*n* = 27,246) vs. 10.31% (*n* = 17,271) (*p* < 0.00001); (iii) aged 45–64 years 32.01% (*n* = 34,092) vs. 24.88% (*n* = 41.668) (*p* < 0.00001); and aged ≥65 years 34.5% (*n* = 36.743) vs. 62.84% (*n* = 105,241) (*p* < 0.00001).

**Figure 6:**
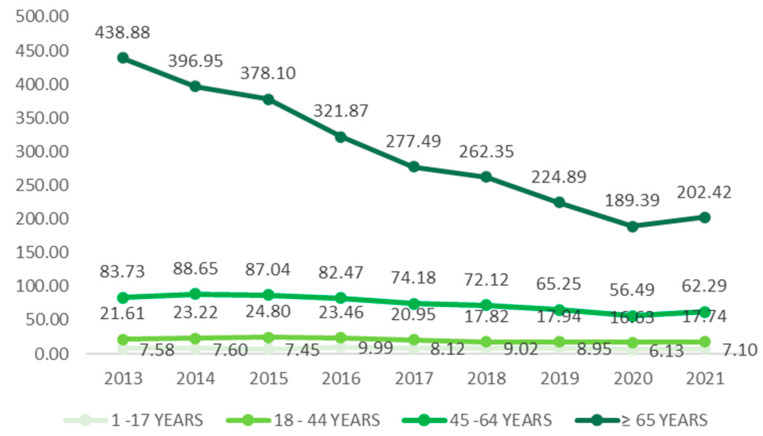
Cases in health care by age group per 100,000 persons.

In Figure 6, the data reveal a high hospitalization rate for cases occurring in any health-care setting, such as nursing homes and hospitals, surpassing the 70% mark. Hospitalization includes admission at the time of the CDI diagnosis or up to seven days after that diagnosis. Remarkably, the age group of 65 years or older shows a lower hospitalization rate in health-care settings, at 63.30%, while in the community, it is the age group with the highest rate, reaching 44.65%. Considering first recurrent cases, it is observed that the fatality rate of CDI is 7.20% in health-care settings, while in the community, this rate is 3.20%. In Figure 6, a significant reduction of 53.87% in the number of cases occurring between 2013 and 2021 in the age group over 65 years in health-care environments stands out. With advanced age being one of the main risk factors for CDI, CDC control from the EIP has been shown to be effective in reducing CDI cases in the older population. In the age group of 45 to 64 years, a significant reduction in the number of cases per 100 thousand inhabitants is also notable, totaling 25.6% of the reduction. In the age group from 18 to 44 years, the reduction was 17.9%. In the younger population, the situation remains stable, with an average of approximately 7 cases for every 100,000 inhabitants.

By gathering fixed and variable costs related to the treatment options for CDI based on information from local hospitals, it was possible to evaluate different treatment scenarios based on days and severity levels (Table 4 and Table 5). After inferring the minimum, maximum, and mean values, the results of the stochastic simulation are presented, utilizing 1000 scenarios with the available treatment options in Brazil. These results are also observed in wards and ICUs (Supplementary Materials).

By simulating the values of treatments for wards and ICUs, it is observed that in wards, the probable value is around USD 2085 (σ ± 182), with a confidence interval (CI) of USD 5.27, while in the ICU, the probable value revolves around USD 4749 (σ ± 439), with a CI of USD 12, as presented in Supplementary Materials. Expenses related to recurrences can be observed, reaching over BRL 240 million, with more than 20.9 thousand cases, resulting in 990 deaths and approximately 2.76% of recurrence cases.

## 4. Discussion

The objective of this study included various stages of maturity, from product validation to its application through a pilot study and a pharmacoeconomic analysis to project the cost–benefit analysis of a potential FMT product. In the first laboratory phase, it was possible to produce a transplantable microbiota with an appropriate bacterial load, as justified by its response in the pilot study, and with minimal residues. The production protocol combined previously described techniques for macromolecule separation, starting with low-speed rotation, followed by high-speed rotation to concentrate the microorganisms [20,21].

A distinguishing feature of the Promicrobioma Project was the development of a lyophilized microbiota, eliminating the need for freezing. This significantly reduces the barriers related to pre-storage, storage, and post-distribution. With the powdered formulation, these factors are eliminated, preventing potential product loss due to inadequate storage, facilitating logistics, and addressing the high cost of frozen medical product logistics in developing countries where hospitals may not have specific areas for this purpose. In our study, FMT showed clinical responses like other transplant studies when indicated for recurrence and clinical failure [22,23,24,25,26,27,28,29,30,31,32]. Success rates for transplants are generally over 80%. Although meta-analyses have demonstrated the success of this therapeutic modality, there are still some questions regarding its widespread use. Until recently, commercially available products were not available, and hospitals relied on microbiota banks. The variability in microbiota used by these banks always raised concerns about potential variability in clinical response, complicating comparisons between studies [33].

FMT is a safe procedure and of significant importance in the treatment portfolio for CDI, considering the limited therapeutic options [34]. The effectiveness of transplantation has a significant impact on patient clinical response, as diarrhea improves within a few days, allowing for rapid patient discharge. This is why we proceeded with a pharmacoeconomic analysis of transplantation in comparison to repeating treatment with conventional drugs.

The severity status of CDI infection has a significant impact on hospital costs and patient management. The guidelines for managing patients with CDI suggest the use of oral vancomycin or fidaxomicin [35,36]. Fidaxomicin is not a drug readily available in most underdeveloped or developing countries, which limits therapeutic options. Additionally, the recurrence rate in severe cases is high, necessitating scarce alternative therapies, which still exhibit a high failure rate. Recent studies have demonstrated a recurrence rate of 21% [37]. Nevertheless, the rate of recurrence may differ based on clinical characteristics including comorbidities and age, as well as the therapy employed. Notably, patients receiving fidaxomicin treatment exhibit a reduced recurrence rate in comparison to those undergoing treatment with oral vancomycin (16% versus 25.4%) [38].

The higher costs naturally occur in moderate and severe cases, or those in intensive care units. Regardless of severity, the costs of CDI are significant for hospitals, estimated at approximately USD 12,470 per patient. Analyzing CDI costs in developing countries is crucial, as studies show cost-effectiveness in various types of interventions, but hospital costs cannot be directly compared across regions with significant economic heterogeneities [39]. Hospital costs in Brazil are much lower compared to developed countries, due to factors such as (1) low remuneration of health-care professionals; (2) outdated and low-cost laboratory tests, excluding molecular biology; (3) generic or similar medications with a risk of lower quality; and (4) poor hospital facilities, with fewer staff for cleaning, maintenance, and patient care. Thus, a hospital stay in Brazil is cheaper, making modern interventions, such as a diagnosis by molecular biology, a new medication, or fecal microbiota transplantation, difficult to achieve cost-effectiveness.

The challenge in comparing cost-effectiveness simulations with other countries lies in determining CDI incidence. In Brazil, CDI is not a notifiable disease, so case frequency is defined by isolated publications [40,41,42,43,44]. In the USA, there has been a notable reduction in cases over the years, even with diagnostic advancements, suggesting that policies on rational use of antimicrobials and contact isolation precautions are effective [45,46]. In Brazil, the disease is underreported as most hospitals lack diagnostic methods, even point-of-care immunochromatographic tests. This underdiagnosis leads to delays in contact isolation, promoting intra-hospital spread. For example, even in Europe, it is estimated that 40,000 patients per year remain undiagnosed due to failure in diagnostic suspicion, despite having point-of-care resources or not [47].

As previously mentioned, fidaxomicin is not available in Brazil, so for the fixed cost simulation, we included only treatment with metronidazole and oral vancomycin, and the cost of fecal microbiota transplantation (FMT). Although FMT is indicated for the treatment of the second recurrence of CDI, trials show that FMT is better even for the first or second episode of CDI [48], i.e., an “early FMT”. Despite such data, the proposal with FMT is to focus its use on the treatment of recurrence. Although FMT in Brazil is relatively expensive, it is much lower than the cost in the USA, where FMT was authorized by the FDA only as a prevention of recurrence [49], even though there is evidence that the therapeutic response based on recurrence with transplantation is superior to a new course with antibiotics [50].

FMT is the most cost-effective treatment, as the way the transplant is administered, whether by colonoscopy, retention enema, nasojejunal, nasogastric, or less invasively, such as the availability of capsules costing between USD 1139 and USD 1946, coupled with rapid diagnostics, has the potential to reduce the average patient stay to 2 or 3 days. There is still debate over the best route for performing FMT, although some studies indicate that the colonoscopy route has lower therapeutic failure [51]. Studies show that infusion through the upper gastrointestinal tract has an efficacy of up to 82% [52], while infusion through the lower gastrointestinal tract has 91.5% [23]. However, given that the risk factors for therapeutic failure of FMT are diverse, such as (i) inflammatory bowel disease, (ii) poor quality of bowel preparation, (iii) previous hospitalization for CDI, and (iv) severe CDI, care must be taken in concluding that there is greater efficacy by a specific route while there are no data with low heterogeneity [29]. Additionally, many variables are linked to cost-effectiveness, and optimizing internal hospital flows can be considered the most cost-effective approach. Therefore, for each strategic decision and hospital action plan, costs will always be related and assumed. The decision to opt for transplantation in cases with moderate recurrence requiring longer treatment, or severe cases, through the study done, is the best decision. In Brazil, regarding treatment options, fecal microbiota transplantation emerges as a more cost-effective alternative compared to antimicrobials when considering direct and indirect costs, but considering only direct costs, transplantation is deemed more cost-effective.

It is important to emphasize that the implementation of FMT requires the availability of adequate infrastructure and qualified professionals for both production and application of the treatment. This study is aligned with global efforts to reduce cases of a bacterium considered an urgent threat, while also illustrating that the adoption of good practices in prescribing, managing, and care leads to positive outcomes, whether in per capita costs, improvement in patient experience, or patient health.

Recognizing *C. difficile*, among other resistant bacteria, as a disease requiring urgent monitoring is crucial to direct resources and attention towards its control and prevention. Encouraging and providing resources to develop the capability of performing fecal microbiota transplants in Brazil is a promising strategy. Ensuring that the most effective treatments are available and accessible to patients is essential. This can include not only fecal transplantation but also updated pharmacological therapies. Balancing the investments of the public and private sectors in health care is an important consideration, as in Brazil health-care spending was 9.23% of the GDP, a value of BRL 914.95 billion, and only 43.03% of this amount refers to state expenditures. It is necessary to evaluate how the public sector can play a more active role in the financing and promotion of health.

## 5. Conclusions

FMT is associated with high success rates in recurrent and refractory CDI using the current product we developed in this project. Furthermore, the FMT is cost-effective, and the procedure should be implemented as routine in institutional protocols. The current product offers an alternative to those available on the market by being a lower-cost option due to its lack of refrigeration requirements. Additionally, its potential superiority compared to antibiotics theoretically reduces the risk of multidrug-resistant bacteria and lowers the likelihood of recurrence. However, further studies are needed, including clinical trials, to confirm these results.

## Figures and Tables

**Table 1:** Patients submitted to fecal microbiota transplant. * Patient admitted in the intensive care unit (ICU) during transplant, FMT—fecal microbiota transplant, DM—diabetes mellitus, SAH—systemic arterial hypertension, HF—heart failure, CRF—chronic renal failure.

Patient	Age	ICU *	Previous Therapy for CDI	Days of Therapy before FMT	Indication of FMT	Mean Daily Evacuations before FMT	Outcome	Clinical Response (Days)	Comorbidities
1	76	No	Vancomycin	42	Recurrence	6	Cure	2	DM, HF, SAH
2	79	No	Vancomycin + metronidazole	28	Recurrence	12	Cure	5	DM, SAH
3	74	No	Vancomycin	36	Recurrence	2	Cure	1	Gastrointestinal neuroendocrinal tumor
4	85	No	Vancomycin	28	Refractory	8	Cure	3	Renal cancer
5	65	No	Vancomycin	14	Recurrence	10	Cure	4	Lung cancer
6	65	No	Vancomycin	5	Refractory	10	Cure	2	Lung cancer
7	38	No	Vancomycin	14	Recurrence	4	Cure	7	Colorectal cancer
8	54	No	Vancomycin	9	Refractory	15	Failure	-	Renal transplant
9	54	No	Vancomycin	10	Refractory	20	Cure	3	Renal transplant
10	67	No	Vancomycin	9	Refractory	8	Cure	5	Chron disease
11	65	No	Vancomycin	15	Refractory	7	Cure	7	-
12	65	No	Vancomycin	14	Recurrence	12	Cure	2	-
13	72	No	Metronidazole	14	Refractory	5	Cure	1	CRF, DM
14	80	Yes	Vancomycin	8	Refractory	6	Cure	1	DM, SAH
15	84	No	Vancomycin	10	Refractory	4	Cure	2	Dementia
16	56	No	Metronidazole	13	Refractory	7	Cure	1	CRF
17	70	Yes	Metronidazole	14	Recurrence	8	Cure	2	DM
18	73	Yes	Vancomycin + metronidazole	28	Refractory	5	Cure	2	DM, stroke
19	53	Yes	Vancomycin + metronidazole	14	Refractory	3	Cure	3	DM, SAH, multiple sclerosis
20	65	Yes	Vancomycin + metronidazole	7	Refractory	12	Cure	1	HIV
21	69	No	Vancomycin + metronidazole	28	Recurrence	6	Cure	3	Stroke, DM, SAH
22	54	Yes	Vancomycin + metronidazole	21	Refractory	8	Cure	5	-
23	56	Yes	Vancomycin + metronidazole	14	Refractory	7	Failure	-	-
24	54	No	Vancomycin	28	Recurrence	5	Cure	7	-

**Table 2:** Total *C. difficile* cases in counties monitored by the CDC. CDI—*Clostridioides difficile* infection. ^1^ Cases per 100,000 persons.

Year	Monitored Population	Community Associated CDI	Community Cases %	Community Cases ^1^	Health-Care-Associated CDI	Health-Care Cases %	Health-Care Cases ^1^
2013	11,552,955	6441	39.32%	55.75	9938	60.68%	86.02
2014	11,533,856	6670	40.84%	57.83	9663	59.16%	83.79
2015	11,682,427	7688	44.30%	65.81	9666	55.70%	82.74
2016	11,777,482	7915	47.12%	67.20	8881	52.88%	75.41
2017	11,906,512	7539	48.60%	63.32	7973	51.40%	66.96
2018	11,982,926	7901	50.68%	65.93	7690	49.32%	64.18
2019	12,058,331	7628	52.20%	63.30	6984	47.80%	57.90
2020	12,104,962	6198	50.55%	51.20	6062	49.45%	50.10
2021	12,109,721	6769	50.71%	55.90	6579	49.29%	54.30

**Table 3:** *Clostridioides difficile* infection simulation cases in Brazil.

Demographic Characteristic	Population ≥ 1 Year of Age	Community-Associated CDI				Health Care-Associated CDI			
		Cases	±σ	Cases 100,000 Persons	±σ	Cases	±σ	Cases 100,000 Persons	±σ
Sex									
Female	99,270,508	69,266	1930	69.77	1.94	94,457	2707	95.15	2.73
Male	103,792,004	41,777	1981	40.25	1.91	69,316	1981	66.78	1.91
Age Group									
1–17 years	45,732,492	8411	238	18.39	0.52	3285	90	7.18	0.20
18–44 years	87,378,489	27,246	774	31.18	0.89	17,271	492	19.77	0.56
45–64 years	46,201,082	34,092	1010	73.79	2.19	41,668	1192	90.19	2.58
65+ years	20,915,569	36,743	1101	175.67	5.27	105,241	3034	503.17	14.5

**Table 4:** Treatment costs based on days spent in wards.

Treatment	Hypothesis (Days)	Fixed Cost Ward USD	Variable Cost Ward USD	Mild USD	Moderate USD	Severe USD
Metronidazole Pill 2 × 250 mg q8h	10	157.81	79.39	1657.43	-	-
	11	157.81	79.62	1815.47	-	-
	12	157.81	79.85	1973.50	-	-
	13	157.81	80.09	2131.54	-	-
	14	157.81	80.32	2289.58	-	-
Vancomycin Ampoule 125 mg q6h	10	157.81	121.54	1699.58	1699.58	-
	11	157.81	125.68	1861.52	1861.52	-
	12	157.81	129.84	2023.49	2023.49	-
	13	157.81	134.02	2185.48	2185.48	-
	14	157.81	138.22	2347.48	2347.48	-
Fecal microbiota transplant	2	157.81	839.54	-	-	1155.15
	3	157.81	839.54	-	-	1312.96

**Table 5:** Treatment costs based on days spent in the intensive care unit (ICU).

Treatment	Hypothesis (Days)	Fixed Cost ICU USD	Variable Cost ICU USD	Mild USD	Moderate USD	Serious USD
Metronidazole Pill 2 × 250 mg q8h	10	382.33	79.39	3902.65	-	-
	11	382.33	79.62	4285.21	-	-
	12	382.33	79.85	4667.77	-	-
	13	382.33	80.09	5050.33	-	-
	14	382.33	80.32	5432.89	-	-
Vancomycin Ampoule 125 mg q6h	10	382.33	121.54	3944.80	3944.80	-
	11	382.33	125.68	4331.27	4331.27	-
	12	382.33	129.84	4717.76	4717.76	-
	13	382.33	134.02	5104.26	5104.26	-
	14	382.33	138.22	5490.79	5490.79	-
Fecal Microbiota Transplant	2	382.33	839.54	-	-	1604.20
	3	382.33	839.54	-	-	1986.52

## Data Availability

Data available upon request.

## Supplementary Materials

The following supporting information can be downloaded at: https://www.mdpi.com/article/10.3390/microorganisms12081741/s1, Table S1: Total *C. difficile* cases in counties monitored by the CDC; Table S2: *C. difficile* simulation cases in Brazil; Table S3: Treatment costs based on days spent in wards; Table S4: Treatment costs based on days spent in the ICU; Table S5: Monte Carlo simulation for the costs of CDI treatment options; Table S6: Probable costs for Inpatient Units and Intensive Treatment Units; Table S7: Recurrence indicators with expenses associated with the number of cases; Figure S1. Microorganisms presented in the Promicrobioma microbiota.
