# Supplementary material for: Validation of Lyophilized Human Fecal Microbiota for the Treatment of Clostridioides difficile Infection: A Pilot Study with Pharmacoeconomic Analysis of a Middle-Income Country—Promicrobioma Project
Source: Microorganisms. 2024 Aug 22;12(8):1741. doi: 10.3390/microorganisms12081741 (PMC11356882; doi:10.3390/microorganisms12081741)
Supplement: Supplementary file 1 [file microorganisms-12-01741-s001.zip › microorganisms-3159186-supplementary.pdf]

SUPPLEMENTARY TABLES AND FIGURE

Table S1: Total *C. difficile* cases in counties monitored by the CDC.

YEAR	MONITORED POPULATION	COMMUNITY ASSOCIATED CDI	COMMUNITY CASES %	COMMUNITY CASES[1]	HEALTHCARE ASSOCIATED CDI	HEALTHCARE CASES %	HEALTHCARE CASES[1]
2013	11,552,955	6,441	39.32%	55.75	9,938	60.68%	86.02
2014	11,533,856	6,670	40.84%	57.83	9,663	59.16%	83.79
2015	11,682,427	7,688	44.30%	65.81	9,666	55.70%	82.74
2016	11,777,482	7,915	47.12%	67.20	8,881	52.88%	75.41
2017	11,906,512	7,539	48.60%	63.32	7,973	51.40%	66.96
2018	11,982,926	7,901	50.68%	65.93	7,690	49.32%	64.18
2019	12,058,331	7,628	52.20%	63.30	6,984	47.80%	57.90
2020	12,104,962	6,198	50.55%	51.20	6,062	49.45%	50.10
2021	12,109,721	6,769	50.71%	55.90	6,579	49.29%	54.30

[1] Cases per 100,000 persons.

Table S2: *C. difficile* simulation cases in Brazil

Demographic Characteristic	Population ≥1 Year of Age	Community Associated CDI				Healthcare Associated CDI			
		Cases	± σ	Cases 100,000 Persons	± σ	Cases	± σ	Cases 100,000 Persons	± σ
**Sex**									
Female	99,270,508	69,266	1,930	69.77	1.94	94,457	2,707	95.15	2.73
Male	103,792,004	41,777	1,981	40.25	1.91	69,316	1,981	66.78	1.91
**Demographic Characteristic**	**Population ≥1 Year of Age**	**Community Associated CDI**				**Healthcare Associated CDI**			
		Cases	± σ	Cases 100,000 Persons	± σ	Cases	± σ	Cases 100,000 Persons	± σ
**Age Group**									
1-17 years	45,732,492	8,411	238	18.39	0.52	3,285	90	7.18	0.20
18-44 years	87,378,489	27,246	774	31.18	0.89	17,271	492	19.77	0.56
45-64 years	46,201,082	34,092	1,010	73.79	2.19	41,668	1,192	90.19	2.58
65+ years	20,915,569	36,743	1,101	175.67	5.27	105,241	3,034	503.17	14.5

Table S3: Treatment costs based on days spent in wards.

Treatment	Hypothesis (days)	Fixed Cost IU US$	Variable Cost IU US$	Mild US$	Moderate US$	Serious US$
Metronidazole Pill 2 × 250 mg 8 h in 8 h	10	157.81	79.39	1,657.43	-	-
	11	157.81	79.62	1,815.47	-	-
	12	157.81	79.85	1,973.50	-	-
	13	157.81	80.09	2,131.54	-	-
	14	157.81	80.32	2,289.58	-	-
Vancomycin Ampoule 125 mg 6 h in 6 h	10	157.81	121.54	1,699.58	1,699.58	-
	11	157.81	125.68	1,861.52	1,861.52	-
	12	157.81	129.84	2,023.49	2,023.49	-
	13	157.81	134.02	2,185.48	2,185.48	-
	14	157.81	138.22	2,347.48	2,347.48	-
Transplant Capsule	2	157.81	839.54	-	-	1,155.15
	3	157.81	839.54	-	-	1,312.96

Table S4: Treatment costs based on days spent in the ICU.

Treatment	Hypothesis (days)	Fixed Cost ITU US$	Variable Cost ITU US$	Mild US$	Moderate US$	Serious US$
Metronidazole Pill 2 × 250 mg 8 h in 8 h	10	382.33	79.39	3,902.65	-	-
	11	382.33	79.62	4,285.21	-	-
	12	382.33	79.85	4,667.77	-	-
	13	382.33	80.09	5,050.33	-	-
	14	382.33	80.32	5,432.89	-	-
Vancomycin Ampoule 125 mg 6 h in 6 h	10	382.33	121.54	3,944.80	3,944.80	-
	11	382.33	125.68	4,331.27	4,331.27	-
	12	382.33	129.84	4,717.76	4,717.76	-
	13	382.33	134.02	5,104.26	5,104.26	-
	14	382.33	138.22	5,490.79	5,490.79	-
Transplant Capsule	2	382.33	839.54	-	-	1,604.20
	3	382.33	839.54	-	-	1,986.52

Table S5: Monte Carlo simulation for the costs of CDI treatment options

IU	Cost US$	σ ±	IC
Metronidazole	1,683	185.71	11.51
Vancomycin	2,325	191.22	11.85
Transplant capsule	1,191	45.71	3.84
**ITU**	**Cost US$**	**σ ±**	**IC**
Metronidazole	4,392	440.82	27.33
Vancomycin	4,550	451.02	27.96
Transplant capsule	9.260	110.61	6.85

Table S6: Probable costs for Inpatient Units and Intensive Treatment Units.

IU		ITU	
**Probable**	**10,428.50**	**Probable**	**23,747.62**
**Average**	9,905.72	**Average**	23,137.87
**Signifcance Level**	0.05	**Signifcance Level**	0.05
**Standard Deviation**	914	**Standard Deviation**	2,196
**Sample**	5,000	**Sample**	5,000
**Confidence Interval**	25.34	**Confidence Interval**	60.87
**Superior Limit**	9,931.06	**Superior Limit**	23,198.74
**Inferior Limit**	9,880.38	**Inferior Limit**	23,077.00
**Minimum**	8,328.00	**Minimum**	19,331.00
**Maximum**	11,502.00	**Maximum**	26,904.00

Table S7: Recurrence indicators with expenses associated with the number of cases.

Recurrence Community	11.98%	12,758	35,784
Recurrence Healthcare	13.75%	23,026	
Community Hospitalization	34.52%	4,404	20,902
Healthcare Hospitalization	71.65%	16,498	
Community Deaths	1.58%	70	990
Healthcare Deaths	5.58%	921	
Recurring expenses IU			217,980,909.63
Recurring expenses ITU			23,514,798.20

Figure S1. Microorganisms presented in the Promicrobioma microbiota

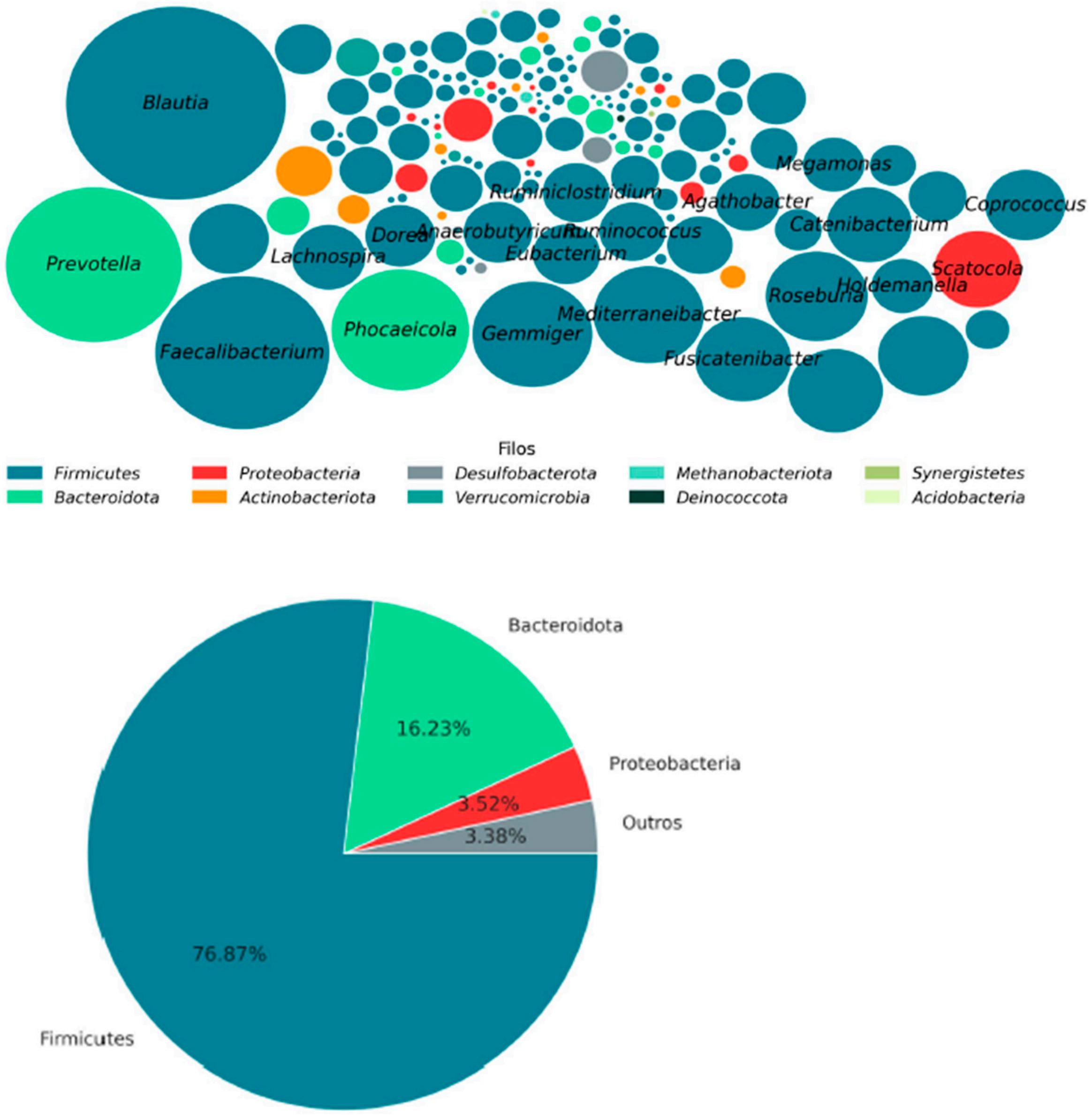

Filo	Classe	Ordem	Família	Gênero	Espécie	%
Firmicutes	Clostridia	Lachnospirales	Lachnospiraceae	Blautia	Blautia wexlerae	10,01
Bacteroidota	Bacteroidia	Bacteroidales	Bacteroidaceae	Prevotella	Prevotella copri	7,03
Firmicutes	Clostridia	Oscillospirales	Ruminococcaceae	Faecalibacterium	Faecalibacterium prausnitzii	6,15
Firmicutes	Clostridia	Oscillospirales	Ruminococcaceae	Gemmiger	-	4,14
Firmicutes	Clostridia	Lachnospirales	Lachnospiraceae	Mediterraneibacter	Mediterraneibacter faecis	3,06
Bacteroidota	Bacteroidia	Bacteroidales	Bacteroidaceae	Phocaeicola	Phocaeicola vulgatus	2,92
Firmicutes	Clostridia	Oscillospirales	Ruminococcaceae	Faecalibacterium	-	2,78
Firmicutes	Clostridia	Lachnospirales	Lachnospiraceae	Fusicatenibacter	Fusicatenibacter saccharivorans	2,67
Firmicutes	Negativicutes	-	-	-	-	2,60
Firmicutes	Negativicutes	Acidaminococcales	Acidaminococcaceae	CAG-266	CAG-266 sp905207485	2,38
Proteobacteria	Alphaproteobacteria	RF32	CAG-239	Scatocola	Scatocola faecipullorum	2,21
Firmicutes	Clostridia	Lachnospirales	Lachnospiraceae	Blautia	-	2,18
Firmicutes	Bacilli	Erysipelotrichales	Coprobacillaceae	Catenibacterium	Catenibacterium sp000437715	2,10
Bacteroidota	Bacteroidia	Bacteroidales	Bacteroidaceae	Prevotella	-	2,06
Firmicutes	Clostridia	Lachnospirales	Lachnospiraceae	Roseburia	Roseburia inulinivorans	2,05
Firmicutes	Clostridia	Lachnospirales	Lachnospiraceae	-	-	1,82
Firmicutes	Clostridia	Lachnospirales	Lachnospiraceae	Blautia	Blautia faecis	1,48
Firmicutes	Clostridia	Oscillospirales	Ruminococcaceae	Ruminiclostridium	Ruminiclostridium siraeum	1,28
Firmicutes	Clostridia	Lachnospirales	Lachnospiraceae	Coprococcus	Coprococcus eutactus	1,26
Firmicutes	Clostridia	Oscillospirales	Ruminococcaceae	-	-	1,22

Filo	Classe	Ordem	Família	Gênero	Espécie	%
Firmicutes	Clostridia	Lachnospirales	Lachnospiraceae	Lachnospira	-	1,14
Firmicutes	Bacilli	Erysipelotrichales	Erysipelotrichaceae	Holdemanella	Holdemanella porci	1,07
Firmicutes	Negativicutes	Selenomonadales	Selenomonadaceae	Megamonas	Megamonas funiformis	1,07
Firmicutes	Clostridia	Lachnospirales	Lachnospiraceae	Dorea	-	1,05
Bacteroidota	Bacteroidia	Bacteroidales	Bacteroidaceae	Phocaeicola	Phocaeicola coprocola	0,97
Firmicutes	Clostridia	Oscillospirales	Acutalibacteraceae	Ruminococcus	Ruminococcus bromii	0,96
Firmicutes	Clostridia	Lachnospirales	Lachnospiraceae	Oliverpabstia	Oliverpabstia intestinalis	0,95
Firmicutes	Clostridia	Lachnospirales	Lachnospiraceae	Roseburia	Roseburia intestinalis	0,94
Firmicutes	Clostridia	Lachnospirales	Lachnospiraceae	Anaerobutyricum	-	0,89
Actinobacteriota	Coriobacteriia	Coriobacteriales	Coriobacteriaceae	Collinsella	-	0,87
Bacteroidota	Bacteroidia	Bacteroidales	Bacteroidaceae	Phocaeicola	Phocaeicola plebeius	0,78
Firmicutes	Bacilli	Erysipelotrichales	Coprobacillaceae	Faecalibacillus	Faecalibacillus intestinalis	0,77
Firmicutes	Clostridia	Lachnospirales	Lachnospiraceae	Acetatifactor	-	0,75
Firmicutes	Clostridia	Lachnospirales	Lachnospiraceae	UMGS1375	UMGS1375 sp900066615	0,70
Proteobacteria	Gammaproteobacteria	Burkholderiales	Burkholderiaceae	Duodenibacillus	Duodenibacillus intestinigallinarum	0,68
Bacteroidota	Bacteroidia	Bacteroidales	Bacteroidaceae	Phocaeicola	Phocaeicola massiliensis	0,67
Firmicutes	Clostridia	Lachnospirales	Lachnospiraceae	Blautia	Blautia obeum	0,67
Firmicutes	Clostridia	Lachnospirales	Lachnospiraceae	Bariatricus	Bariatricus comes	0,65
Desulfobacterota	Desulfovibrionia	Desulfovibrionales	Desulfovibrionaceae	-	-	0,62

Filo	Classe	Ordem	Família	Gênero	Espécie	%
Firmicutes	Clostridia	Oscillospirales	Butyricicoccaceae	Agathobaculum	Agathobaculum butyriciproducens	0,61
Firmicutes	Clostridia	Lachnospirales	Lachnospiraceae	Anaerostipes	Anaerostipes hadrus	0,61
Firmicutes	Clostridia	Lachnospirales	Lachnospiraceae	Agathobacter	Agathobacter faecis	0,60
Firmicutes	Clostridia	Lachnospirales	Lachnospiraceae	Coprococcus	Coprococcus catus	0,59
Firmicutes	Clostridia	Lachnospirales	Lachnospiraceae	Agathobacter	Agathobacter rectalis	0,58
Firmicutes	Bacilli	Lactobacillales	Streptococcaceae	Streptococcus	Streptococcus thermophilus	0,58
Firmicutes	Clostridia	Lachnospirales	Lachnospiraceae	Ventrimonas	-	0,53
Verrucomicrobia	Verrucomicrobiae	Verrucomicrobiales	Akkermansiaceae	Akkermansia	Akkermansia muciniphila	0,52
Firmicutes	Clostridia	Lachnospirales	Lachnospiraceae	Copromonas	Copromonas sp000435795	0,51
Firmicutes	Clostridia	Oscillospirales	Acutalibacteraceae	Eubacterium	-	0,49
Firmicutes	Clostridia	Christensenellales	CAG-74	UBA11524	UBA11524 sp000437595	0,47
Firmicutes	Clostridia	Lachnospirales	Lachnospiraceae	Clostridium	Clostridium fessum	0,46
Firmicutes	Clostridia	Lachnospirales	Lachnospiraceae	Anaerobutyricum	Anaerobutyricum hallii	0,45
Firmicutes	Clostridia	Lachnospirales	Lachnospiraceae	Eubacterium	Eubacterium ramulus	0,41
Firmicutes	Clostridia	Lachnospirales	Lachnospiraceae	Eisenbergiella	Eisenbergiella sp900066775	0,40
Firmicutes	Clostridia	Peptostreptococcales	Peptostreptococcaceae	-	-	0,37
Bacteroidota	Bacteroidia	Bacteroidales	Bacteroidaceae	Bacteroides	Bacteroides uniformis	0,35
Firmicutes	Clostridia	Lachnospirales	Lachnospiraceae	Copromonas	-	0,35
Firmicutes	Clostridia	Oscillospirales	Oscillospiraceae	Evtepia	Evtepia gabavorous	0,34

Filo	Classe	Ordem	Família	Gênero	Espécie	%
Firmicutes	Clostridia	Oscillospirales	Oscillospiraceae	-	-	0,34
Firmicutes	Clostridia	Lachnospirales	Lachnospiraceae	Dorea	Dorea formicigenerans	0,34
Firmicutes	Clostridia	Lachnospirales	Lachnospiraceae	Mediterraneibacter	Mediterraneibacter torques	0,32
Firmicutes	Clostridia	Lachnospirales	Lachnospiraceae	Lachnospira	Lachnospira eligens	0,28
Firmicutes	Clostridia	Lachnospirales	Lachnospiraceae	Choladocola	Choladocola sp003480725	0,28
Proteobacteria	Alphaproteobacteria	RF32	CAG-239	HGM16780	HGM16780 sp900538765	0,28
Firmicutes	Clostridia	Oscillospirales	Oscillospiraceae	CAG-83	CAG-83 sp001916855	0,27
Firmicutes	Clostridia	Oscillospirales	Acutalibacteraceae	CAG-177	CAG-177 sp900770255	0,27
Firmicutes	Clostridia	TANB77	CAG-508	CAG-269	CAG-269 sp003525075	0,25
Firmicutes	Clostridia	Oscillospirales	Ruminococcaceae	Ruminococcus	Ruminococcus sp000433635	0,25
Firmicutes	Clostridia	UBA1381	UBA1381	CAG-41	CAG-41 sp900066215	0,25
Firmicutes	Clostridia	Lachnospirales	Lachnospiraceae	Acetatifactor	Acetatifactor sp900066565	0,23
Desulfobacterota	Desulfovibrionia	Desulfovibrionales	Desulfovibrionaceae	Bilophila	Bilophila wadsworthia	0,23
Firmicutes	Clostridia	TANB77	CAG-508	Merdicola	Merdicola sp900552655	0,23
Firmicutes	Bacilli	Lactobacillales	Streptococcaceae	Streptococcus	-	0,22
Actinobacteriota	Coriobacteriia	Coriobacteriales	Eggerthellaceae	Slackia	Slackia isoflavoniconvertens	0,22
Firmicutes	Clostridia	Lachnospirales	Lachnospiraceae	Choladocola	Choladocola sp003481535	0,20
Firmicutes	Clostridia	Lachnospirales	Lachnospiraceae	Eubacterium	Eubacterium sp003491505	0,20
Bacteroidota	Bacteroidia	Bacteroidales	Barnesiellaceae	Barnesiella	Barnesiella intestinihominis	0,20

Limite of 0.2%
